# Improved Performance Fiber Bragg Grating Hydrogen Sensor Based on Pt/WO_3_ Nanosheets and Nafion Hybrid Coatings

**DOI:** 10.3390/nano16100637

**Published:** 2026-05-21

**Authors:** Wenhui Zhou, Hongxiao Li, Jinyu Zhang, Jixiang Dai, Wenbin Hu, Cheng Cheng, Minghong Yang

**Affiliations:** 1 National Engineering Research Center for Fiber Optic Sensing Technology and Networks, Wuhan University of Technology, Wuhan 430070, China; 15871280139@163.com (W.Z.); lihongxiao@whut.edu.cn (H.L.); 18972997218@163.com (J.Z.); wenbin_hu@whut.edu.cn (W.H.); cheng@whut.edu.cn (C.C.); minghong.yang@whut.edu.cn (M.Y.); 2 State Key Laboratory of Maritime Technology and Safety, Wuhan University of Technology, Wuhan 430063, China

**Keywords:** fiber Bragg grating, Pt-loaded WO_3_ coating, capillary, hydrogen sensor

## Abstract

Reliable detection of hydrogen leakage is essential for the safe operation of hydrogen-related facilities. In this work, we propose a compact fiber Bragg grating (FBG) hydrogen sensor that exhibits high sensitivity. The sensor is based on an FBG encapsulated in a capillary, deposited with a hybrid coating of Pt/WO_3_ nanosheets and Nafion, which can effectively prevent the detachment of sensitive materials and facilitate mass production. The optimized sensor exhibits a wavelength shift of 1383 pm and a response time of 16 s towards 1% H_2_ in air at room temperature, outperforming other FBG hydrogen sensors. In addition, the sensor displays nearly linear response and good repeatability during the hydrogen exposure process. Furthermore, the response of the sensor to hydrogen is much higher than that of other reducing gases. Nevertheless, more than 80% of the sensitivity of this sensor can be maintained even in 85% humidity atmosphere. This work presents an effective strategy to improve the performance of FBG hydrogen sensors, which can promote their potential application for hydrogen detection.

## 1. Introduction

Hydrogen serves as a critical feedstock and energy carrier in diverse fields, including in petrochemicals, organic chemical synthesis and aerospace. At ambient temperature and pressure, hydrogen is a colorless, odorless and highly flammable gas. Hydrogen facilities have the potential to explode upon exposure to an ignition source, as hydrogen has a wide explosive range (4.0% to 75.6% by volume) in air [1,2,3,4]. Consequently, safely detecting hydrogen leakage is critically important for taking emergency measures to avoid potential explosions. According to the classification of sensing signals, hydrogen sensors can be divided into electrochemical and optical types. Compared to electrochemical sensors [5], optical hydrogen sensors have obvious merits, such as lower risks of electrical spark generation and good anti-electromagnetic interference capability [6,7]. Most optical hydrogen sensors consist of optical fiber and hydrogen-sensitive materials, and their working principles are based on measuring variations in optical signals. Based on detecting principles, these fiber-optic hydrogen sensors are categorized as micro-mirror sensors [8], interferometric sensors [9,10], surface plasmon resonance sensors [11], and fiber Bragg grating (FBG) sensors [12,13]. Compared to other optical hydrogen sensors, FBG hydrogen sensors have obvious advantages, such as distributed measurement capability and ease for temperature compensation.

FBG sensors are widely used due to their immunity to electromagnetic interference and multiplexing capability [14,15]. Conventional FBG hydrogen sensors are prepared by coating hydrogen-sensitive materials directly onto the surface of the grating section [16]. The thermal interaction between the sensitive material and hydrogen causes red shifts in the central wavelength of FBG, enabling hydrogen concentration detection [17]. In 1999, Sutapun et al. [18] reported an FBG hydrogen sensor based on Pd thin films. This sensor is more suitable for detecting hydrogen in an oxygen-free environment. Therefore, preparing a hydrogen sensor for hydrogen leakage detection in the air is more meaningful, as most hydrogen facilities are installed in this atmosphere. Several processing methods, such as side-polishing FBGs [19], and femtosecond laser etching technology [20] have been employed to improve the sensitivity of FBG hydrogen sensors. Although the sensitivity of these sensors can be improved, the stability of the sensing device is reduced due to the degradation of the mechanical properties of FBG. To solve this problem, preparing a highly sensitive coating is more feasible for improving the performance of hydrogen sensors.

However, several critical challenges still need to be solved to meet the demand of practical application. Direct coating of hydrogen-sensitive materials onto the bare FBG surface is an approach offering high sensitivity and fast response due to direct thermal contact. However, this method suffers from coating detachment [21] due to different thermal expansion coefficients between the coating and the fiber [22]. In addition, the physical changes in the coating can exert mechanical stress on the optical fiber, leading to deformation of the grating section [23]. These negative effects can cause signal drift of the FBG, thus the stability and accuracy of the FBG hydrogen sensor will be reduced.

To overcome these issues, various encapsulation strategies have been explored. Quartz grooves and a titanium substrate were used as a substrate to load the FBG and the hydrogen-sensitive material for hydrogen sensing [24,25]. However, this structure reduces the hydrogen diffusion channels, leading to a slow response rate of the hydrogen sensor. In addition, the sensitivity of the hydrogen sensor was sacrificed as most thermal energy was conducted to the substrate.

In this paper, we propose a compact structure consisting of a capillary coated with annealed Nafion and Pt/WO_3_ nanosheets, which significantly enhances the performance of hydrogen sensors. The sensing principle of this FBG hydrogen sensor is based on an exothermic reaction upon hydrogen exposure [26]. The temperature sensitivity of FBG is about 10 pm/°C, which is similar to that of standard FBG [27]. By keeping the sensing coating away from the FBG, our capillary-encapsulated structure can conduct the thermal energy efficiently, while minimizing the mechanical stress interference on conventional directly coated FBG hydrogen sensors. Based on the reported thermal decomposition process of Nafion [28], its decomposition degree can be adjusted by setting an appropriate annealing temperature. The critical role of noble metal nanoparticles in enhancing gas-sensing performance has been extensively established. For example, gold nanoparticles decorated on carbon nanofibers have been shown to achieve an increase in response to ethanol, along with a faster response time and improved stability [29]. In our work, Pt nanoparticles serve a similar function for hydrogen sensing. WO_3_ is functionalized with a Pt catalyst [30], which accelerates the reaction between the catalyst and hydrogen, enabling a reliable response to hydrogen in air [31]. The sensitivity of the hydrogen sensor is closely associated with the morphology of WO_3_ materials, as it provides an active site for Pt catalysts.

## 2. Experimental

**Figure 1:**
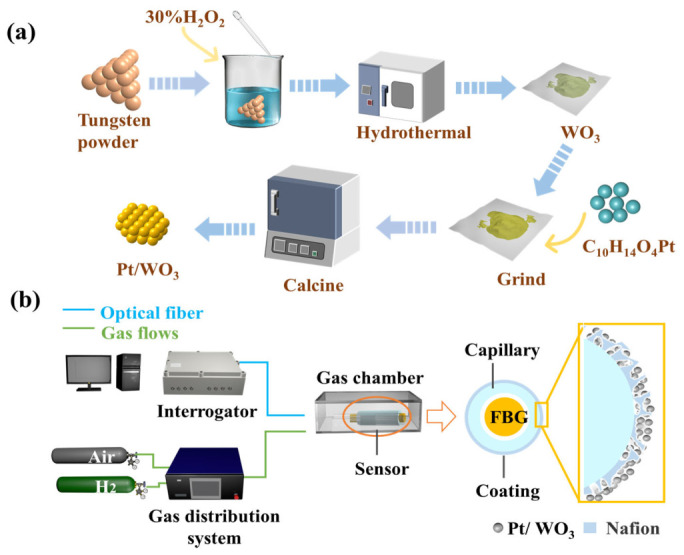
(**a**) Diagram of Pt/WO_3_ preparation process; (**b**) Diagram of FBG hydrogen sensing system.

As illustrated in Figure 1a, a hydrogen-sensitive material was synthesized via a hydrothermal method. Firstly, 3 g tungsten powder was dispersed in deionized water, and 15 mL 30% H_2_O_2_ was added under stirring to form WO_3_ precursor. Then, the precursor was hydrothermally treated at 200 °C for 6 h in a furnace. After being washed and dried, the sample was mixed with platinum acetylacetonate (molar ratio W:Pt = 10:1), and the mixture was calcined at 350 °C to obtain Pt/WO_3_ powder.

The prepared powder was subsequently mixed with Nafion solution (5 wt% in water) to form a uniform slurry, which was coated onto the capillary surface and annealed at different temperatures. The quartz capillary (outer diameter 550 μm, inner diameter 300 μm, length 15 mm), made of high-purity silica, offers excellent chemical inertness, thermal stability, and mechanical strength. It can operate at high temperatures (1000 °C) in an atmosphere and resist corrosion from most chemicals except hydrofluoric acid, thus serving as a protective element. Finally, the coated capillaries were assembled onto the grating region of the FBG to prepare hydrogen sensors.

In this sensing setup, a reference FBG is employed for temperature compensation. This FBG is fabricated with the same process as the sensing FBG but without the hydrogen-sensitive coating, and is placed inside the capillary adjacent to the sensing FBG. It effectively reduces the influence of ambient temperature fluctuations, thereby providing temperature compensation.

Figure 1b illustrates the FBG hydrogen sensing system. The sensors are placed in a gas chamber and connected to an FBG demodulator. Various concentrations of hydrogen are injected into the gas chamber via a gas distribution system. Wavelength shifts in FBG resulting from the exothermic reaction between the sensitive coating and hydrogen are monitored in real time by the demodulator and recorded by a computer.

## 3. Results and Discussions

### 3.1. Characterization of the Sensing Material

**Figure 2:**
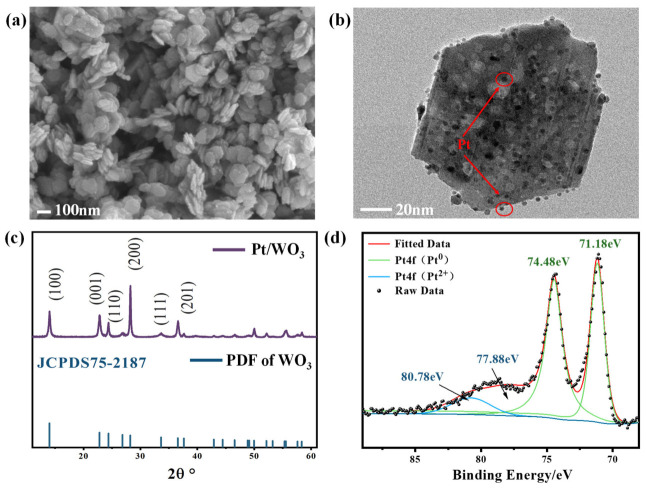
(**a**) SEM pattern; (**b**) TEM pattern, and (**c**) XRD pattern of the Pt/WO_3_ sample. (**d**) XPS pattern of the Pt element of the Pt/WO_3_ sample.

Figure 2a,b show the SEM and TEM images of Pt/WO_3_ powder. The sensitive material exhibits plate morphology with a diameter of about 100 nm, with Pt nanoparticles uniformly dispersed on the surface. Figure 2c presents the XRD pattern of the hydrogen-sensitive material. The diffraction peaks of the sensitive material match well with those of hexagonal-phase WO_3_ (JCPDS #75-2187), confirming the successful synthesis of WO_3_ via the hydrothermal method. Figure 2d shows the XPS pattern of the Pt element of the Pt/WO_3_ sample. The Pt4f spectrum was deconvolved into two doublets corresponding to the Pt4f_7/2_ and Pt4f_5/2_ spin–orbit components. The dominant doublet at 71.18/74.48 eV is assigned to Pt^0^, while the minor doublet at 77.88/80.78 eV is assigned to Pt^2+^, which may originate from surface oxidation of Pt or incomplete decomposition of the platinum acetylacetonate precursor.

**Figure 3:**
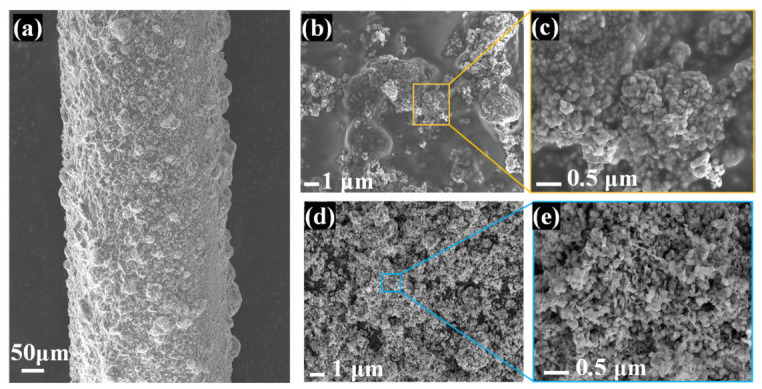
(**a**) SEM pattern of sensitive coating deposited on a capillary. SEM pattern of capillary surface coating (**b**,**c**) before; (**d**,**e**) after 375 °C annealing process.

Figure 3a shows the SEM images of the coating deposited on the capillary. The coating of the hydrogen-sensitive material mixed with Nafion (annealed at 375 °C) is evenly distributed on the capillary surface, fully encapsulating the capillary. To investigate the effect of annealing temperature on the microstructure of the hydrogen-sensitive coatings, SEM was employed to observe the sensitive coating on the capillary surface before and after calcination. Surface morphologies before the annealing process are presented in Figure 3b,c. Figure 3b shows a relatively dense surface with some Pt/WO_3_ clusters. Higher magnification images reveal that these clusters are stacked Pt/WO_3_ nanosheets. As shown in Figure 3d,e, the surface of the sensitive coating exhibits a looser structure with more pores generated after the 375 °C annealing process. In addition, some Pt/WO_3_ particles can be observed to transform into plate-like shapes with clear edges and a diameter of approximately 100 nm. The annealed Nafion can form a network structure coating around the quartz capillary. Therefore, it can serve as an intermediate carrier to enhance bonding strength between the hydrogen-sensitive coating and the quartz capillary. In addition, the annealed Nafion can anchor the Pt/WO_3_ nanosheets and prevent their agglomeration, which can enhance the stability of the FBG hydrogen sensor.

**Figure 4:**
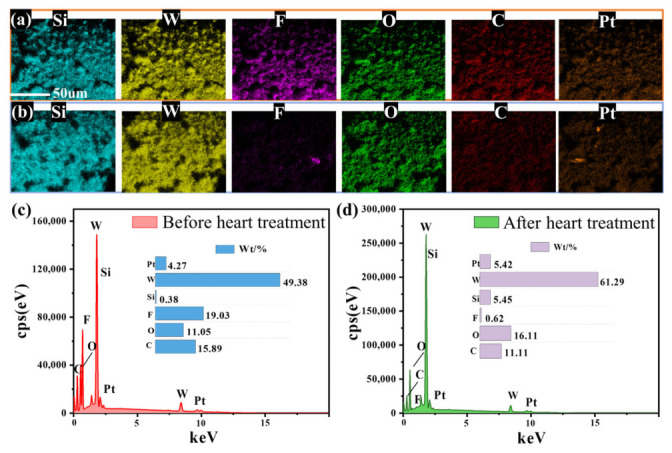
The EDS mappings of the sensitive coating on the capillary (**a**) before; (**b**) after annealing process. Elemental survey spectrum of sensitive coating on capillary (**c**) before; (**d**) after annealing process.

Energy-dispersive spectroscopy (EDS) analysis was performed on the coating of the hydrogen-sensitive material mixed with Nafion solution coated on the capillary. The results are shown in Figure 4a,b. The Pt element in the coating is uniformly distributed together with W and O elements, while Si, C, and F elements are also evenly dispersed on the coating. Therefore, the Nafion membrane uniformly wraps the capillary and encapsulates the sensitive material. However, a significant reduction in the F and C elements on the surface of the coating was observed after the thermal treatment, further proving the decomposition of the fluorinated polymer Nafion. Furthermore, the Pt element remained relatively stable before and after heat treatment. These results confirm that the annealing process does not lead to clear detachment or loss of the Pt catalyst, thereby ensuring the integrity of the catalytic function of the sensitive material. According to the elemental analysis (Figure 4c,d), the reduction in F and C elements can also be verified. The mass ratio of W to Pt was about 11.30:1 after the 375 °C annealing process. This ratio is slightly higher than the theoretical raw material ratio of 9.42:1, which may be attributed to partial overlap between the characteristic peaks of W and those of Si from the quartz substrate, leading to a systematic overestimation of the measured W content. The decrease in the C element after the annealing process can be attributed to the decomposition of Nafion.

### 3.2. Hydrogen Sensing Performance

To investigate the effect of annealing temperature on sensing performance, FBG hydrogen sensors annealed at different temperatures were placed in a gas chamber for hydrogen sensing. In this work, these sensors, deposited with sensitive coatings as prepared, annealed at 350 °C, 375 °C, and 425 °C, were labeled as FBG1, FBG2, FBG3, and FBG4 hydrogen sensors, respectively. These annealing temperatures were selected based on the thermal decomposition of Nafion [28,32,33] to prepare different sensing components. Their sensing performance was first evaluated by exposing these sensors to 1% H_2_. In this work, the response time was calculated as the wavelength shift in FBG reaching 90% of its maximum value. The response and recovery characteristics of four sensors were tested at 1% H_2_ with air as carrier gas.

**Figure 5:**
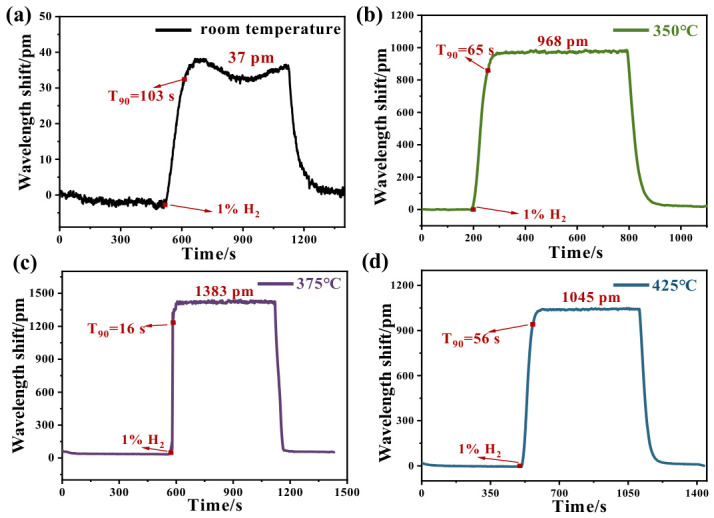
Response of FBG hydrogen sensor with constant concentration (1% H_2_) of (**a**) room temperature; (**b**) annealed at 350 °C; (**c**) annealed at 375 °C; (**d**) annealed at 425 °C.

As shown in Figure 5a, the FBG hydrogen sensor based on the as-deposited coating shows a wavelength shift in only about 37 pm in 1% H_2_, with relatively larger signal fluctuations. This is mainly because the unannealed Nafion membrane completely encapsulates the Pt/WO_3_ particles, forming a dense organic-inorganic composite coating. Such a structure hinders the diffusion of hydrogen molecules, allowing the gas to contact only a small number of active sites. The sensing coating calcined at 350 °C exhibits a wavelength shift of 968 pm and a response time of 65 s when exposed to 1% H_2_ (Figure 5b). This temperature corresponds to the decomposition and volatilization of the sulfonic acid side groups of Nafion. From Figure 5c, the sensor based on a coating calcined at 375 °C shows the best performance, with a wavelength shift of up to 1383 pm and a response time down to 16 s. At this temperature, the sulfonic acid groups of Nafion can be decomposed during the annealing process. Based on the above sensitivity value (10 pm/°C), the relationship between the thermal effect and the wavelength shift can be calculated. Thus, the measured temperature rise is about 138 °C during 1% hydrogen exposure. Sensitive coating annealed at 425 °C (as shown in Figure 5d) shows slightly lower sensitivity, which may be due to the growth of Pt catalyst nanoparticles [34].

**Figure 6:**
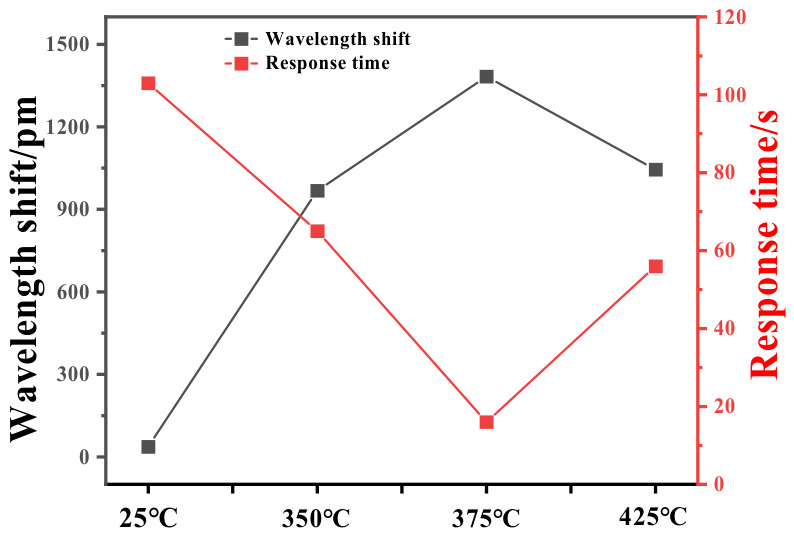
Response performance of FBG hydrogen sensors annealed at different temperatures.

Figure 6 shows the response trend of these sensors annealed at different temperatures. It can be seen that the sensitivity and response rate of the FBG hydrogen sensor gradually improve with the increase in annealing temperature, reaching the best sensitivity and quickest response rate at 375 °C. Then, the sensing performance reduces when the annealing temperature is increased to 425 °C. As the sensitive coating annealed at 375 °C shows the best performance, further experiments were carried out by employing the FBG3 hydrogen sensor.

**Figure 7:**
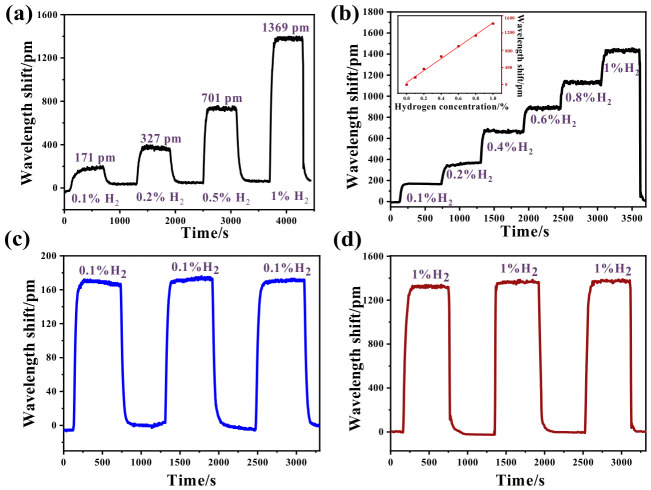
Response of FBG3 hydrogen sensor towards (**a**) different concentrations of hydrogen; (**b**) increasing concentrations of hydrogen; (**c**) three cycles of 0.1% H_2_; (**d**) three cycles of 1% H_2_.

To further investigate the performance of the FBG3 hydrogen sensor, the gas chamber was sequentially filled with hydrogen at various concentrations. As displayed in Figure 7a, the hydrogen sensor exhibits wavelength shifts of 171 pm, 327 pm, 701 pm, and 1369 pm when exposed to 0.1% H_2_, 0.2% H_2_, 0.5% H_2_, and 1% H_2_, respectively. From Figure 7b, the wavelength shift in the hydrogen sensor exhibits a corresponding stepwise increase as the hydrogen concentration increases gradually within the range of 0~1% H_2_. It can be clearly observed that the hydrogen sensor shows an approximately linear wavelength shift in response to varying hydrogen concentrations. This characteristic is crucial for practical hydrogen monitoring applications, as it indicates a predictable relationship between wavelength shift and hydrogen concentration, thereby facilitating reliable hydrogen monitoring. Figure 7c shows three cycles exposure of 0.1% H_2_. The sensor maintains a wavelength shift of around 174 pm over three cycles. As shown in Figure 7d, the FBG hydrogen sensor displays a wavelength shift of approximately 1370 pm during three cycles exposure of 1% H_2_, demonstrating its good repeatability for hydrogen exposure.

**Figure 8:**
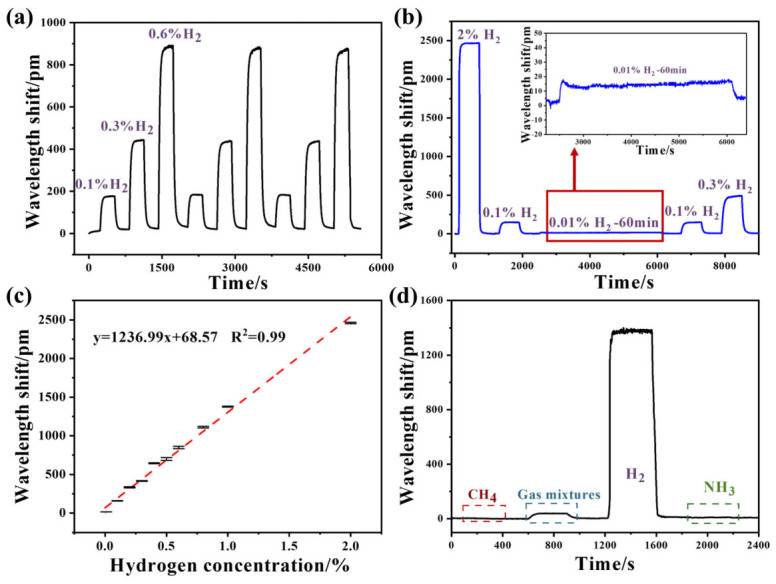
Response of FBG3 hydrogen sensor towards (**a**) three cycles of 0.1%, 0.3%, and 0.6% H_2_; (**b**) different hydrogen concentrations. Response of FBG3 hydrogen sensor towards (**c**) different hydrogen concentrations (0~2% H_2_); (**d**) different gases.

To better verify the stability of the hydrogen sensor, multiple-cycle hydrogen tests were conducted at different concentrations. Firstly, three cycles of hydrogen exposure at 0.1%, 0.3%, and 0.6% were carried out in this work. As shown in Figure 8a, the sensor exhibits upward wavelength shifts with an increase in hydrogen concentrations. Wavelength shifts in FBG3, a hydrogen sensor, are nearly the same under the same concentration of hydrogen, indicating its good repeatability. Then, a larger concentration range from 0.01%~2% H_2_ exposure was performed in the following experiment (Figure 8b). The sensor can show a quick response towards 2% H_2_ in air, with a wavelength shift of about 2460 pm and little hysteresis in this process. When exposed to a low concentration of 0.01% H_2_, the sensor shows a considerable wavelength shift of approximately 16 pm during 60 min hydrogen exposure. Based on a demodulator resolution of 1 pm (≈6.3 ppm H_2_), and a typical wavelength fluctuation of 2~3 pm in air (≈19 ppm H_2_), the detection limit of the optimized sensor is calculated as 20 ppm. Overall, this sensor displays favorable recovery characteristics during various hydrogen exposure processes, demonstrating good responsivity to hydrogen detection. This is of great significance for achieving stable and reliable hydrogen monitoring in practical applications.

As shown in Figure 8c, the sensor exhibits an approximately linear response to increasing hydrogen concentrations from 0.01% to 2%. The error bars represent fluctuations of wavelength shift after the hydrogen response essentially reaches equilibrium. Theoretically, the resolution of the optimized FBG3 hydrogen sensor can be as low as 9 ppm within this hydrogen concentration range. Based on the fitting results, the coefficient of determination (R^2^) is 0.99, and the slope varies from 1203.6 to 1270.38. The FBG3 hydrogen sensor shows high sensitivity at room temperature, which is beneficial for early warning of hydrogen leakage. This excellent performance indicates that the sensor is capable of monitoring hydrogen over a wide concentration range, demonstrating its robust capability in hydrogen detection.

The hydrogen sensor was sequentially exposed to 1% CH_4_ (air as the carrier gas) and to a gas mixture consisting of 5.04% CO_2_, 4.98% C_2_H_4_, 5.03% C_2_H_2_, and 5.04% C_2_H_6_. Subsequently, 1% H_2_ was introduced to the gas room for hydrogen sensing. At last, 0.1% NH_3_ was introduced into the gas room for demonstrate the selectivity of this sensor. During this process, the hydrogen concentration is much higher than in other mixed gases. However, wavelength shifts caused by hydrogen are significantly greater than those caused by other interference gases, confirming its good selectivity for hydrogen sensing. Compared to the reported work [35], this sensor shows an obvious improvement in responsivity, with approximately 10% higher sensitivity and a 30% faster response rate towards 1% H_2_. These improvements can be mainly attributed to the plate-like nanostructure of the sensitive coating, which facilitates faster hydrogen diffusion and provides more active sites.

**Figure 9:**
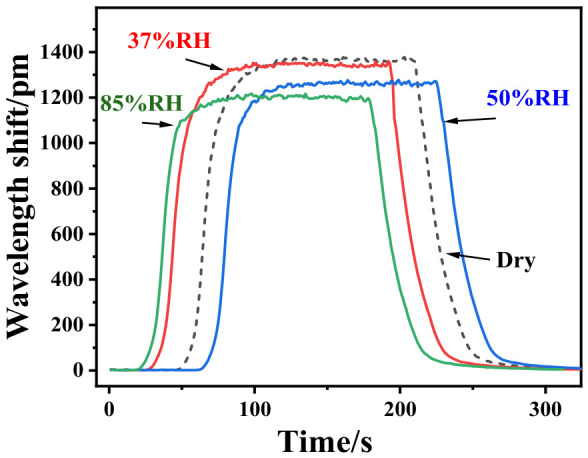
Hydrogen response under different relative humidity (RH).

To assess the sensor’s performance under humid conditions, the FBG3 hydrogen sensor towards 1% hydrogen was conducted at different relative humidities (Figure 9). As more active sites of sensitive coating are covered by water molecules, the FBG3 hydrogen sensor shows lower sensitivity under a higher-humidity atmosphere. This humidity dependence is likely due to water adsorption on residual polar groups of the partially decomposed Nafion [36].

**Figure 10:**
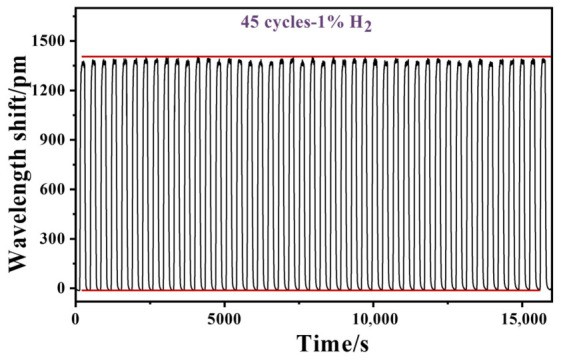
Response of the FBG hydrogen sensor towards 45 cycles of 1% H_2_.

Long-term stability was further evaluated by performing 45 consecutive cycles of exposure to 1% H_2_ at room temperature. As shown in Figure 10, the average maximum wavelength shift over the 45 cycles is around 1385 pm. The baseline drift remains within ±10 pm throughout the hydrogen sensing process, and no significant sensitivity decay can be observed. These results demonstrate the good stability and repeatability of the capillary-encapsulated sensor, which is crucial for practical hydrogen monitoring applications.

In addition, the proposed capillary encapsulation structure facilitates the large-scale fabrication of hydrogen-sensing probes. Unlike conventional deposition of sensitive materials on each FBG, this preparation method separates the fabrication of the sensing element from the inscription of the Bragg gratings. Thus, it can avoid damage to the FBG during the high-temperature annealing process. Multiple capillaries deposited with hydrogen-sensitive coatings can be sequentially loaded on FBGs with different wavelengths, enabling a more flexible preparation method for distributed hydrogen sensing. Table 1 shows the performance comparison of recently reported FBG hydrogen sensors. Among these sensors, the sensor in this work exhibits the fastest response time of 16 s and the highest wavelength shift of 1383 pm toward 1% H_2_.

Despite these advantages, the proposed design has some limitations. The first limitation is its limited operating temperature, as annealed Nafion can decompose in a high-temperature environment. Another drawback is the high cost of the sensing system due to the employ of noble Pt as a catalyst, which limits its massive application. Moreover, the mechanical robustness of the capillary structure under an alkaline environment still needs further investigation. In addition, future research can also focus on the following points to improve the performance of hydrogen sensors. Firstly, employing a hydrogen-sensitive coating with hydrophobic properties may be an effective method to improve the sensing performance of FBG hydrogen sensors. Secondly, using a flexible hydrogen-sensitive coating without Pt (such as Fe-N/C) has great potential to reduce the cost of the distributed sensing systems. Finally, it is more feasible to achieve a distributed hydrogen sensing system utilizing optical fiber integrated with optical frequency domain reflectometry (OFDR) technology.

## 4. Conclusions

This study proposes a compact FBG hydrogen sensor employing a capillary encapsulation structure. Material characterization confirms the successful hydrothermal synthesis of hexagonal nanosheet WO_3_, and the hybrid coating of annealed Nafion and Pt/WO_3_ nanosheet is uniformly distributed on the surface of the capillary, enabling robust integration of the active material with the FBG. An FBG hydrogen sensor with a coating annealed at 375 °C can exhibit a wavelength shift of 1383 pm and a rapid response time of 16 s under 1% hydrogen atmosphere. The sensor also displays nearly linear response and good repeatability during the hydrogen exposure process, with a low detection limit of 0.01% H_2_ (≈16 pm wavelength shift). This linearity is beneficial for hydrogen monitoring in practical applications. Moreover, this sensor shows good selectivity toward hydrogen, further verifying its applicability in complex atmospheres. In summary, the proposed capillary-encapsulated FBG hydrogen sensor offers high sensitivity, fast response, and good selectivity, making it a good candidate for distributed hydrogen concentration monitoring in various scenarios.

## Figures and Tables

**Table 1:** Comparison of recently reported FBG hydrogen sensors.

Publication Year, Reference	Response Time and Sensitivity	Materials, Methods
2017, [12]	20~30 s, 530 pm (1% H_2_)	Pt/WO_3_, hydrothermal method
2022, [16]	34 s, 110 pm (1% H_2_)	TBAOH-Pt/WO_3_, polymer intercalated method
2024, [24]	165 s, 572 pm (1% H_2_)	Porous Pt/WO_3_, MOF template method
2024, [35]	25~30 s, 1200 pm (1% H_2_)	H-Pt/WO_3_, sol–gel method
2025, [13]	595 s, 258 pm (4% H_2_)	Pd_78_Ag_15_Ni_7_, magnetron sputtering technique
This work	16 s, 1383 pm (1% H_2_)	Nafion/Pt/WO_3_, annealed at 375 °C

## Data Availability

The original contributions presented in this study are included in the article. Further inquiries can be directed to the corresponding author.
